# Genome-wide identification of the pea protein Phosphatase 2C (PP2C) family and expression patterns during abiotic stress and seed development

**DOI:** 10.1186/s12870-026-08768-4

**Published:** 2026-07-14

**Authors:** Guoxing Xue, Daming Ouyang, Lincao Song, Haizhu Yang, Ailing He, Huan Li, Chengpeng Wu, Quanzhi Zhao, Jingjun Ruan

**Affiliations:** College of Agriculture, Guizhou University, Guiyang, Guizhou China

**Keywords:** *Pisum sativum*, *PP2C* gene family, Abiotic stress, Seed development, Expression pattern

## Abstract

**Background:**

Protein phosphatase 2C (PP2C), a member of the largest family of phosphatases in plants, plays a crucial role in regulating stress response, growth and development, and hormone signaling. The pea (*Pisum sativum*) is globally cultivated legume crop of significant for both food and forage. In agricultural production, abiotic stresses such as drought, soil salinity, and extreme temperature severely limit pea yield. Although the *PP2C* gene family is known to be critically involved in growth regulation and abiotic stress response, no systematic studies on this gene family in peas have been reported to date.

**Results:**

In this study, 93 pea *PP2C* genes were identified on a genome-wide level and designated *PsPP2C1* through *PsPP2C93* according to their chromosomal locations. The 93 PsPP2C proteins were analyzed for parameters including amino acid sequence length, molecular weight, isoelectric point, predicted subcellular localization, exon number, and hydrophilicity. The pea *PP2C* gene family was divided into 13 subfamilies (A–L) and four tandem duplication and 23 segmental duplication events were identified. The expression levels of 20 selected *PsPP2C* genes were investigated under four types of abiotic stress and across different seed development stages. *PsPP2C15* exhibited strong upregulation under all four stress conditions, with expression increasing more than 13-fold under cold stress. In contrast, expression of *PsPP2C1* and *PsPP2C58* was suppressed across all stress treatments. During seed development, *PsPP2C7* showed notably high expression, while *PsPP2C15* was highly expressed in 21-day-old leaves. Subcellular localization predictions indicated that *PsPP2C15* is likely chloroplast-localized. Promoter analysis revealed an enrichment of cis-acting elements associated with photosynthesis and hormone regulation, suggesting that *PsPP2C15* may play an important role in both developmental processes and abiotic stress responses in pea.

**Summary:**

This study presents the first genome-wide identification and characterization of the *PP2C* gene family in pea, comprising 93 members. The expression patterns of 20 *PsPP2C* genes were analyzed by quantitative real-time PCR (qRT-PCR), under abiotic stresses and during seed development, providing insights into their potential biological functions. Those findings suggest that the PP2C family is important for pea growth and development, with PsPP2C15 emerging as a key candidate for further investigation in abiotic stress tolerance and seed development.

**Supplementary Information:**

The online version contains supplementary material available at https://doi.org/10.1186/s12870-026-08768-4.

## Background

*Pisum sativum* L. is an important winter legume crop cultivated globally for both human consumption and animal fodder [1]. Major pea-producing regions include Canada, China, Russia, India, and the United States [2]. Like other legumes, pea seeds are nutritionally dense, containing high levels of proteins, carbohydrates, vitamins, and minerals. The rich protein content positions pea-derived plant proteins as a viable alternative to animal-based proteins [3]. Notably pea seeds are abundant in lysine but limited in sulfur-containing amino acids, a profile complementary to that of cereals, which are typically lysine-deficient but contain adequate sulfur amino acids [4]. Consequently, incorporating peas into cereal-based diets can enhance overall amino acid balance. In agricultural systems, abiotic stresses such as drought, flooding, salinity, and temperature extremes significantly limit the productivity of grain and legume crops. Among these, drought is a predominant factor adversely affecting crop yield and quality [5]. Prolonged drought conditions often exacerbate soil salinity, further imposing salt stress on plants.

Abscisic acid (ABA) is a key phytohormone involved in regulating plant growth, development, and adaptation to environmental cues. Research since its discovery has established the PYL–PP2C–SnRK2 signaling module as the core ABA transduction pathway [6, 7]. Protein phosphatase 2C (*PP2Cs*) genes are evolutionarily conserved and present across prokaryotes and eukaryotes [8, 9]. Reversible phosphorylation, a critical post-translational modification governing cellular signaling and development, is coordinated by protein kinases (PKs) and protein phosphatases (PPs) [10, 11]. While PKs phosphorylate serine (Ser), threonine (Thr), and tyrosine (Tyr) residues, PPs catalyze dephosphorylation [12]. Based on their substrate specificity and catalytic mechanisms, PPs can be classified into three groups: serine/threonine phosphatases (STPs), protein tyrosine phosphatases (PTPs), and dual-specificity phosphatases (DSPTPs) [13, 14]. PTPs are further divided into phosphoprotein metallophosphatases (PPMs) and phosphoprotein phosphatases (PPPs). PP2C belong to the PPM family, require Mg^2+^/Mn^2+^cofactors, and function as essential regulators in signal transduction pathways in plants [15].

In plants, the PP2C family represents the largest group of phosphatases. These proteins typically contain a conserved catalytic domain at either the N- or C-terminus, while the opposite terminus exhibits low sequence conservation, contributing to functional diversification [14–16]. PP2C participate broadly in plant physiology, including hormone signaling—such as in ABA and salicylic acid (SA) pathways [17, 18], and in responses to abiotic stresses like drought [19], cold [20], and salinity [21]. Extensive studies in *Arabidopsis* have revealed functional specialization among PP2C subfamilies. Subfamily A: negative regulators of ABA signaling [22]; subfamily B: associated with mitogen-activated protein kinases (MAPK) signaling [23]; subfamily C: involved in flower development [24, 25]; subfamily D: related to salt stress response [26]; subfamily E: modulated stomatal signaling [27]; and subfamily F: regulated plant defense mechanisms [28]. Beyond *Arabidopsis*, the *PP2C* gene family has been identified and characterized in multiple species, including wheat [29], maize [30], rice [31], and soybean [32], underscoring its conserved and pivotal role in plant growth, development, and stress adaptation.

Although the *PP2C* gene family has been identified and studied in a wide range of plants, there has been no systematic analysis of the pea *PP2C* gene family. This study aims to identify and characterize *PP2C* genes in pea and explore their expression under abiotic stress and seed development.

## Results

### Identification and bioinformatic analysis of the PP2C family in peas

Based on the whole pea genome, pea *PP2C* candidate genes were identified using the BLASTp method. After removing duplicate entries and genes lacking the PP2C catalytic domains through domain validation, a final set of 93 pea *PP2C* genes were obtained. These genes were designated *PsPP2C1* through *PsPP2C93* according to their chromosome positions and the Latin name of the pea. Bioinformation analyses were performed on the 93 corresponding PsPP2C proteins, including amino acid sequence length, molecular weight, isoelectric point, predicted subcellular localization, exon number, and grand average hydropathicity (Table S1).

The amino acid sequence length of PP2C family members ranged from 96 to 1136 residues, with molecular weights spanning 11.24 to 120.34 kDa. The isoelectric points varied from 3.83 to 9.66, while the instability index ranged from 21.94 to 65.43, and the aliphatic index from 67.25 to 93.52 (Table S1).

The grand average of hydropathicity values fell between − 0.612 (PsPP2C32) and 0.06 (PsPP2C58), confirming that all PsPP2Cs were hydrophilic proteins. Subcellular localization predictions indicated that most *PsPP2C* genes were localized in the nucleus, cytoplasm, and chloroplasts. A smaller subset was predicted to localize to mitochondria, vesicles, or the cytoskeleton, suggesting that *PsPP2C* genes may perform diverse functions, and play distinct roles throughout pea growth and development.

### Classification of the *PsPP2C* gene and construction of its protein phylogenetic tree

**Fig. 1:**
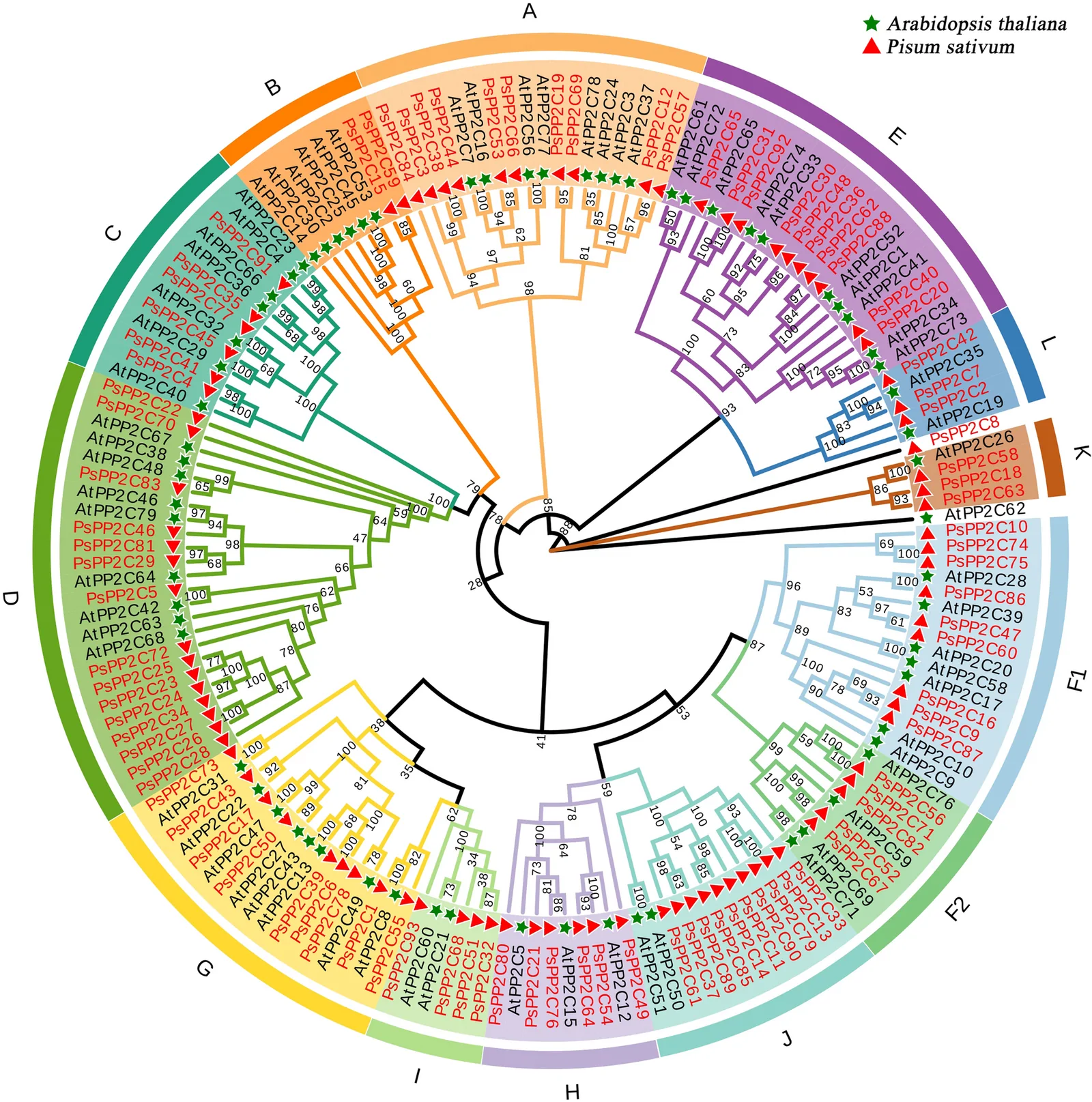
Phylogenetic tree of PP2C proteins in the pea and Arabidopsis. Red triangles represent the pea and green asterisks represent Arabidopsis. In the phylogenetic tree, the node numbers are bootstrap values, the red font is for pea PP2C proteins and the black font is for Arabidopsis PP2C proteins. Different subgroups correspond to different region colors

To investigate the evolutionary relationships of the *PP2C* gene family in pea, a phylogenetic tree was constructed using the neighbor-joining (NJ) method with 1,000 bootstrap replicates. The analysis included 93 pea PP2C proteins and 70 *Arabidopsis* PP2C proteins (Table S2; Fig. 1). Following the established *Arabidopsis* classification system [31], the pea *PP2C* family was categorized into 13 subgroups (A–L). Subgroup D contained the highest members, with 15 PsPP2C protein. Subgroups A, E, and J, each comprised 10 members, followed by subgroups F1 and G with 9 members each. Subgroups C and H both contained 6 members each, subgroup F2 had 4, subgroup I had 4, and subgroups K and L each included 3 members. Subgroup B was the smallest, consisting of only 2 PsPP2C proteins. Notably, PsPP2C8 frequently formed a distinct branch within the phylogenetic tree, suggesting that it may play a unique functional role in pea growth and development.

### Gene structure, conserved motifs, and promoter cis-acting element analysis of the *PsPP2C* gene family

**Fig. 2:**
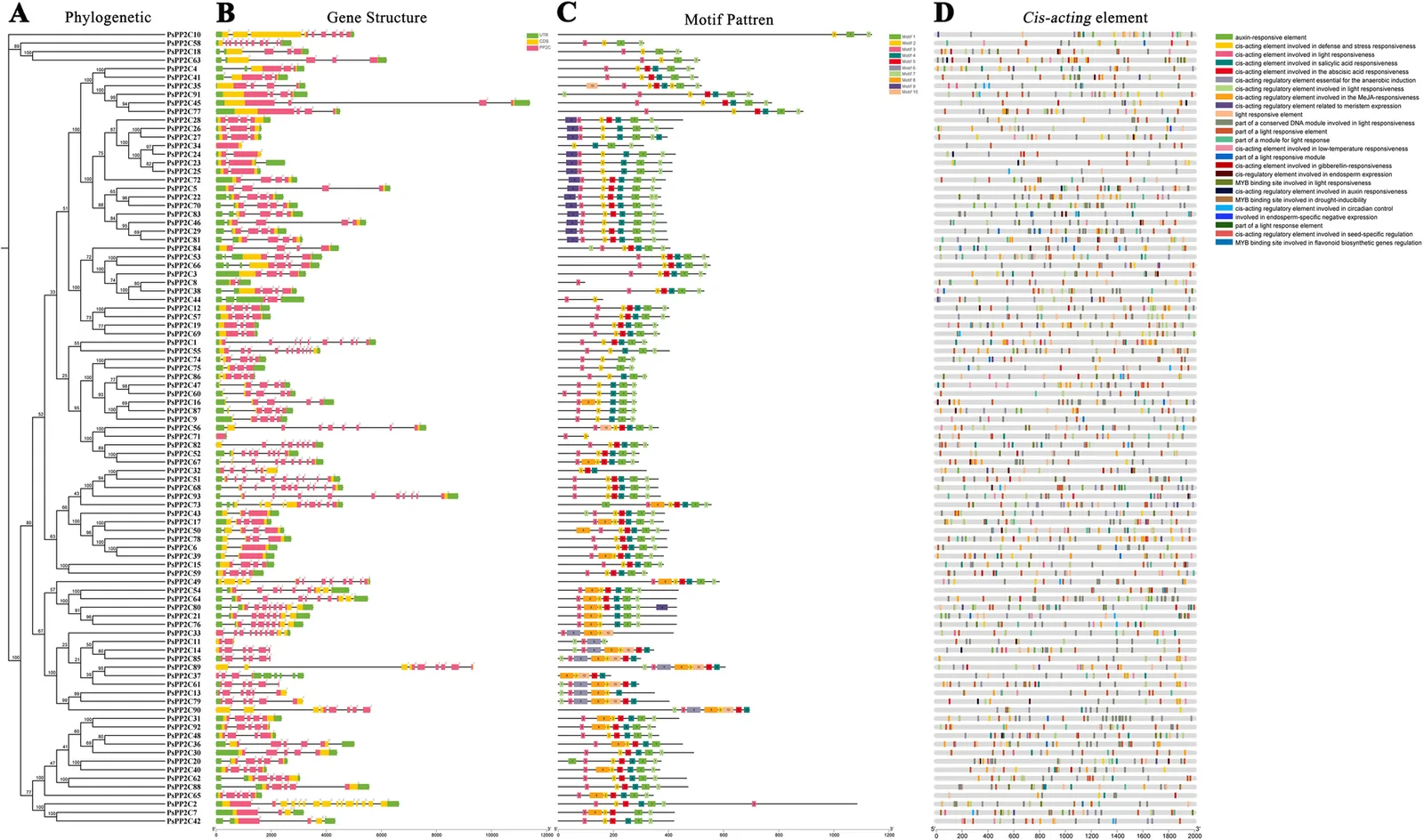
Phylogenetic relationships, gene structures, motif distributions, and cis-acting elements of the 93 *PsPP2C* gene families. **A** Phylogenetic tree of pea PP2C proteins; node numbers are bootstrap values. **B** Schematic diagram of the gene structure of the 93 *PsPP2C* genes for the translated region (UTR), Coding sequence (CDS), structural domains, and introns, with the number indicating the phase of the corresponding intron. **C** Amino acid conserved motifs in the 93 PsPP2C proteins (motif 1–10); the black line indicates the relative length of the corresponding protein. **D** Cis-acting elements of the 2000 bp promoter sequence upstream of the 93 *PsPP2C* genes; colored blocks correspond to different cis-acting elements

The exon/intron architecture of genes and the diversity of protein structural domains are important factors in the evolution of gene families. A phylogenetic tree was constructed with 1000 bootstrap replicates using the full-length protein sequences of the 93 PsPP2Cs, and the corresponding gene structures, motif compositions, and promoter cis-acting elements were analyzed (Fig. 2; Table S3). The phylogenetic tree analysis revealed that the PsPP2C proteins within the same subgroup are evolutionarily closely and cluster together on the tree (Fig. 2A). In terms of gene structure, *PsPP2C* genes belonging to the same subgroup had generally share similar exon/intron numbers, despite variation in exon or intron length (Fig. 2B; Table S1). For instance, all members of subgroup B contain four exons, and all members of subgroup C also have four exons, except for *PsPP2C4*, which possesses five exons. Subgroups A, D, E, F1, F2, and I likewise exhibit largely consistent—or only minimally differing—exon/intron counts, with occasional exceptions among certain members.

To investigate the conserved motifs of PsPP2C proteins and their evolutionary relationships, 10 conserved motifs of PsPP2C proteins were analyzed using the online MEME website (https://meme-suite.org/meme/tools/MEME) (Fig. 2C; Table S3). Most of the PsPP2C proteins contain the conserved motifs 1, 2, 3, 4, 5, and 7 and the gene structures and the composition of the conserved motifs in the same subgroup are highly similar, and there are some differences in the gene structures and conserved motifs between different subgroups.

To investigate the potential functions of the *PsPP2C* genes, cis-acting element analyses were conducted on the 2 kb promoter region upstream of all 93 *PsPP2C* genes (Fig. 2D; Table S3). The results revealed a variety of important *cis*-acting elements within these promoters. These include elements associated with hormone responses—such as auxin, methyl jasmonate, abscisic acid (ABA), salicylic acid (SA), and gibberellin —as well as elements related to stress tolerance and growth processes. Among the latter are motifs responsive to low-temperature, drought, defense and stress, endosperm- or seed-specific expression. Notably, light-responsive elements were particularly abundant in the *PsPP2C* promoters. Together, these findings suggest that the *PsPP2C* genes likely play significant roles in pea growth and development and are closely linked to light-mediated regulation.

### Distribution of *PsPP2C* genes on chromosomes and gene fragment replication

**Fig. 3:**
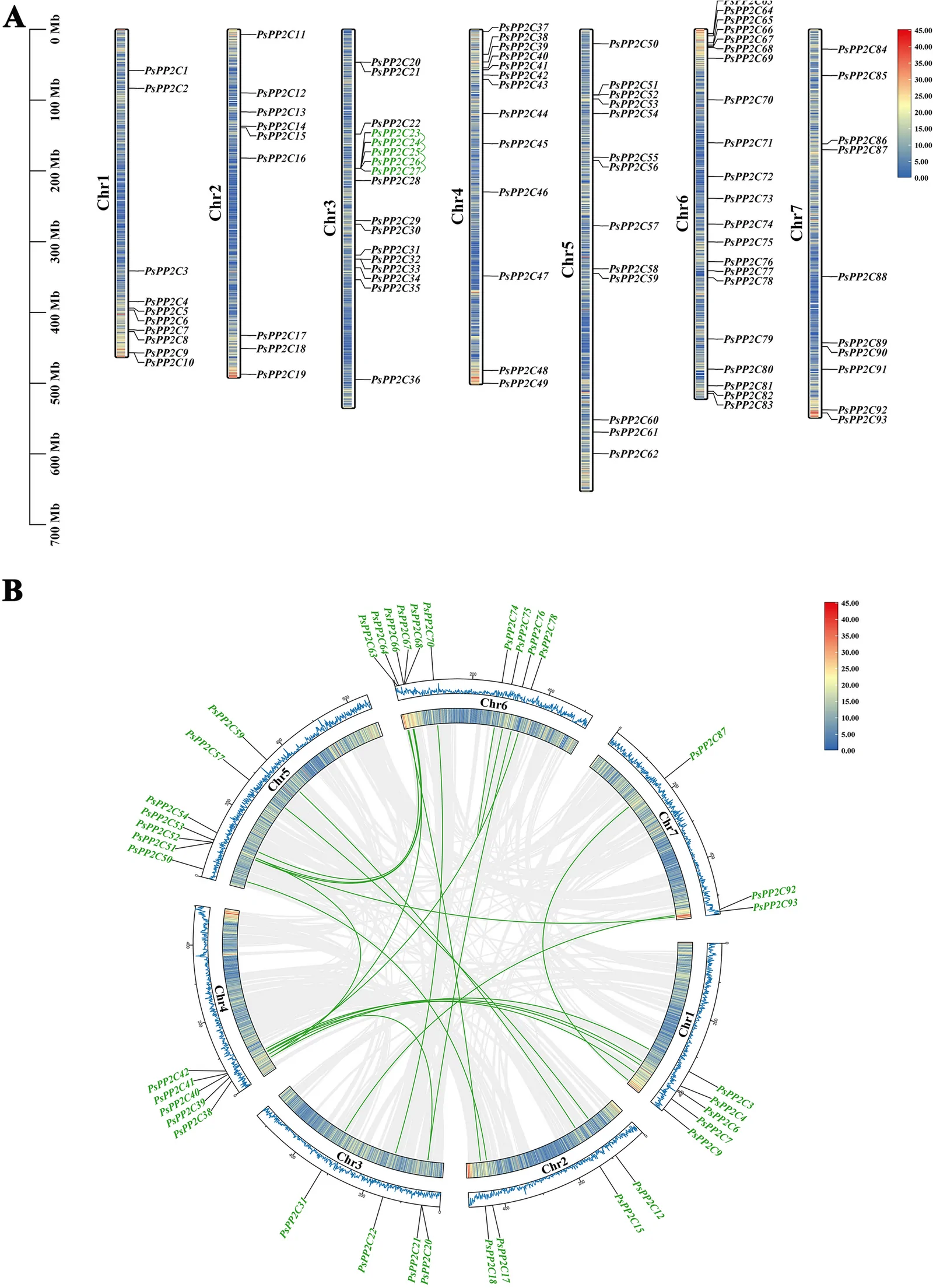
Distribution of *PsPP2C* genes on chromosomes and the gene replication relationship (**A**) Distribution of 93 *PsPP2C* genes on chromosomes; green font represents tandem repeat sequences; on chromosomes is the gene density (Bin Size = 1,000,000), from blue to red indicating the number of genes contained in that length interval from less to more, respectively. **B** Distribution of the 23 *PsPP2C* genes on chromosomes and gene replication relationships. Grey lines represent gene pairs between different chromosomes and green lines represent *PsPP2C* gene pairs. From inside to outside, the first one is the chromosome, and the chromosome information is consistent with (**A**). The second outer circle is the chromosome gene density (Bin Size = 1,000,000); the higher the fold, the greater the gene density

Tandem duplication and segmental replication events play important roles in gene amplification and the generation of novel gene functions. The distribution of the *PsPP2C* gene on chromosomes and duplication pattern of the *PsPP2C* gene family (Fig. 3; Table S4). The 93 *PsPP2C* genes were unevenly distributed across the seven pea chromosomes (Fig. 3A). The highest number of genes was located on Chr6, followed by 17 (~ 18.28%) on Chr3, 13 (~ 13.98%) each on Chr4 and Chr5, and 10 (~ 10.75%) each on Chr1 and Chr7. Chr2 Contained the fewest genes, with only nine (~ 9.68%). Within the *PsPP2C* family, four tandem duplication events (Fig. 3A; Table S4) and 23 segmental duplication events (Fig. 3B; Table S4) were identified. The tandem duplications events occurred on Chr3 and involved five genes: *PsPP2C23*, *PsPP2C24*, *PsPP2C25*, *PsPP2C26*, and *PsPP2C27*. Four of these genes all belonging to subgroup D, participated in tandem duplication events. Segmental duplications were distributed across all seven chromosomes, and each subgroup contained at least one segmentally duplicated gene. These results indicate that both tandem and segmental duplications contribute significantly to the expansion of the *PsPP2C* genes family, with segmental duplications playing a more prominent role overall. However, in subgroup D, tandem duplications appear to have contributed more to gene amplification than segmental duplications.

### Evolutionary analysis of the *PP2C* family of peas in relation to *PP2C* families of other species

**Fig. 4:**
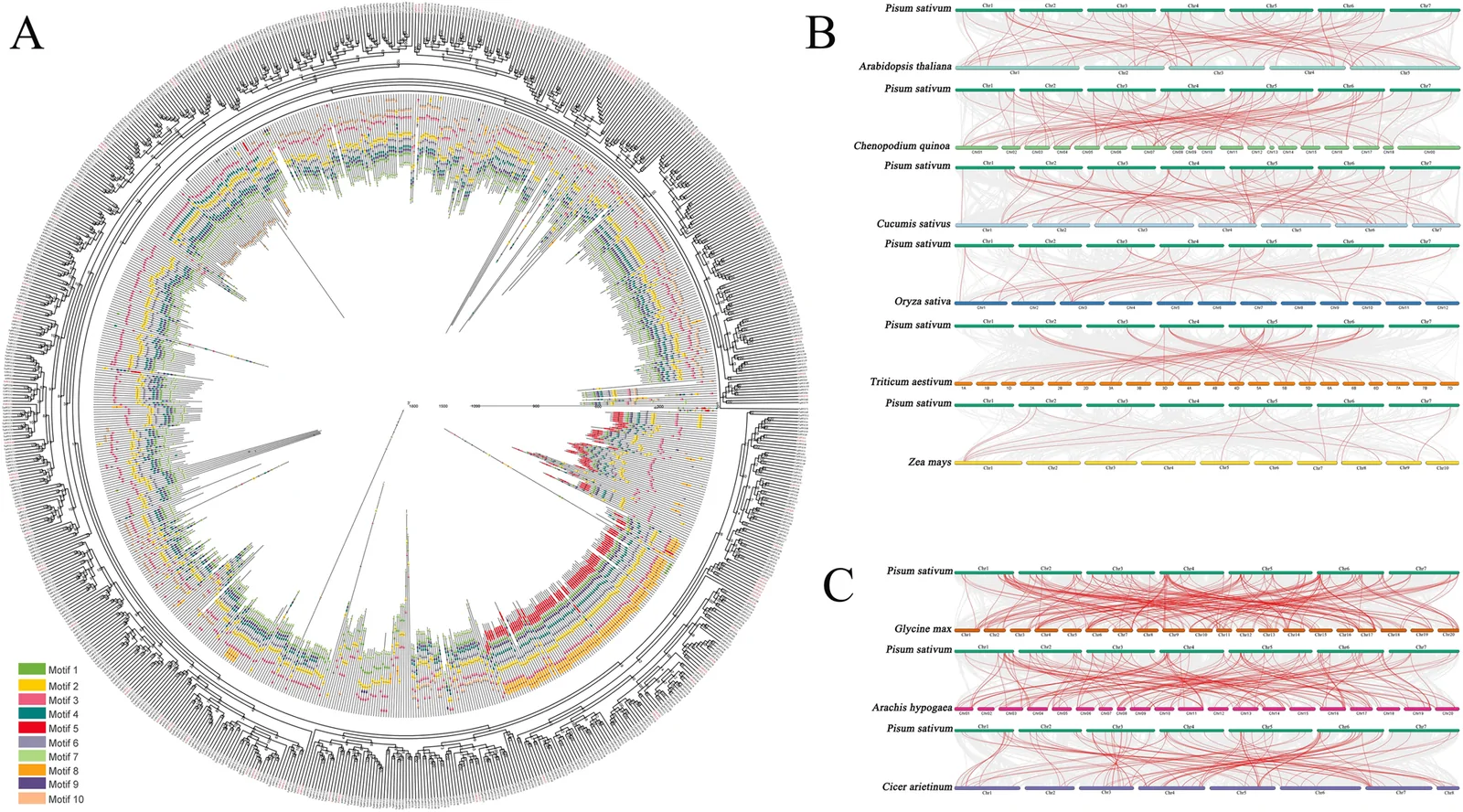
Phylogenetic tree, conserved motif composition and covariance analysis of PP2C in pea and six other plants (**A**) Phylogenetic tree and conserved motif composition of PP2C proteins in pea and six plants (*Arabidopsis thaliana*, *Chenopodium quinoa*, *Cucumis sativus*, *Oryza sativa*, *Triticum aestivum*, and *Zea mays*); node numbers are bootstrap values, pea PP2C protein is labelled in red, different module colors represent different conserved motifs. **B** The collinearity analysis of the *PP2C* gene between peas and six plant species (*A. thaliana*, *C. quinoa*, *C. sativus*, *O. sativa*, *T. aestivum*, and *Z. mays*); the red line represents the collinear region of the *PP2C* gene between peas and this species, the gray line represents the collinear region of the genome between peas and the indicated species. **C** The collinearity analysis of *PP2C* between peas and three leguminous plants (*Glycine max*, *Cicer arietinum*, and *Arachis hypogaea*); the red line represents the collinear region of the *PP2C* gene between peas and this species; the gray line represents the collinear region of the genome between peas and this species

To investigate the evolutionary relationships of the PP2C family across species, a phylogenetic tree was constructed using PP2C protein sequences from pea and six other species: three dicots (*Arabidopsis thaliana*, *Chenopodium quinoa* and *Cucumis sativus*) and three monocots (*Oryza sativa*, *Triticum aestivum*, and *Zea mays*). Conserved protein motifs were analyzed using the MEME suite (https://meme-suite.org/meme/tools/MEME) (Fig. 4A; Table S5). Furthermore, synteny analyses were performed between the pea PP2C family and six species, as well as between pea and three legume species (*Glycine max*, *Cicer arietinum*, and *Arachis hypogaea*) (Fig. 4B and C; Table S6). The phylogenetic analysis revealed that most of PsPP2C proteins clustered together and formed independent branches separate from dicot PP2C, with very few PsPP2Cs grouping with monocot PP2Cs. Conserved motifs analysis showed that motifs 1, 2, 3, 4, 6, and 9 were present in majority of PP2C proteins. Analysis of the homologous genes pairs indicated that *PsPP2C* genes shared the highest number of homologous pairs with *G. max* (211 pairs), followed by *A. hypogaea* (156 pairs), *C. arietinum* (93 pairs), *C. quinoa* (83 pairs), *C. sativus* (66 pairs), *A. thaliana* (60 pairs), *T. aestivum* (42 pairs), *O. sativa* (26 pairs) and *Z. mays* (15 pairs) (Table S6).

Comparative analysis showed that the *PsPP2C* genes had the greatest number of homologous pairs with legumes, followed by dicots, and the fewest with monocots. Some *PsPP2C* genes exhibited homology with genes from monocot, dicot, and legume species (e.g., *PsPP2C12*, *PsPP2C15*, *PsPP2C64*, and *PsPP2C78*). Others were homologous only to dicot and legume species (e.g., *PsPP2C5*, *PsPP2C16*, *PsPP2C21*, PsPP*2C52*, *PsPP2C60*, and *PsPP2C68*), while a subset showed homology exclusively with legume species (e.g. *PsPP2C36* and *PsPP2C82*). These results suggest that certain *PsPP2C* genes may have originated in ancestors common to dicots and monocots prior to their divergence. Subsequent differentiation within dicot ancestors,, following their separation from monocots, likely gave rise to further specialized *PP2C* genes, with some evolving into legume-specific forms.

### Tissue-specific expression pattern of the *PsPP2C* gene under abiotic stress

**Fig. 5:**
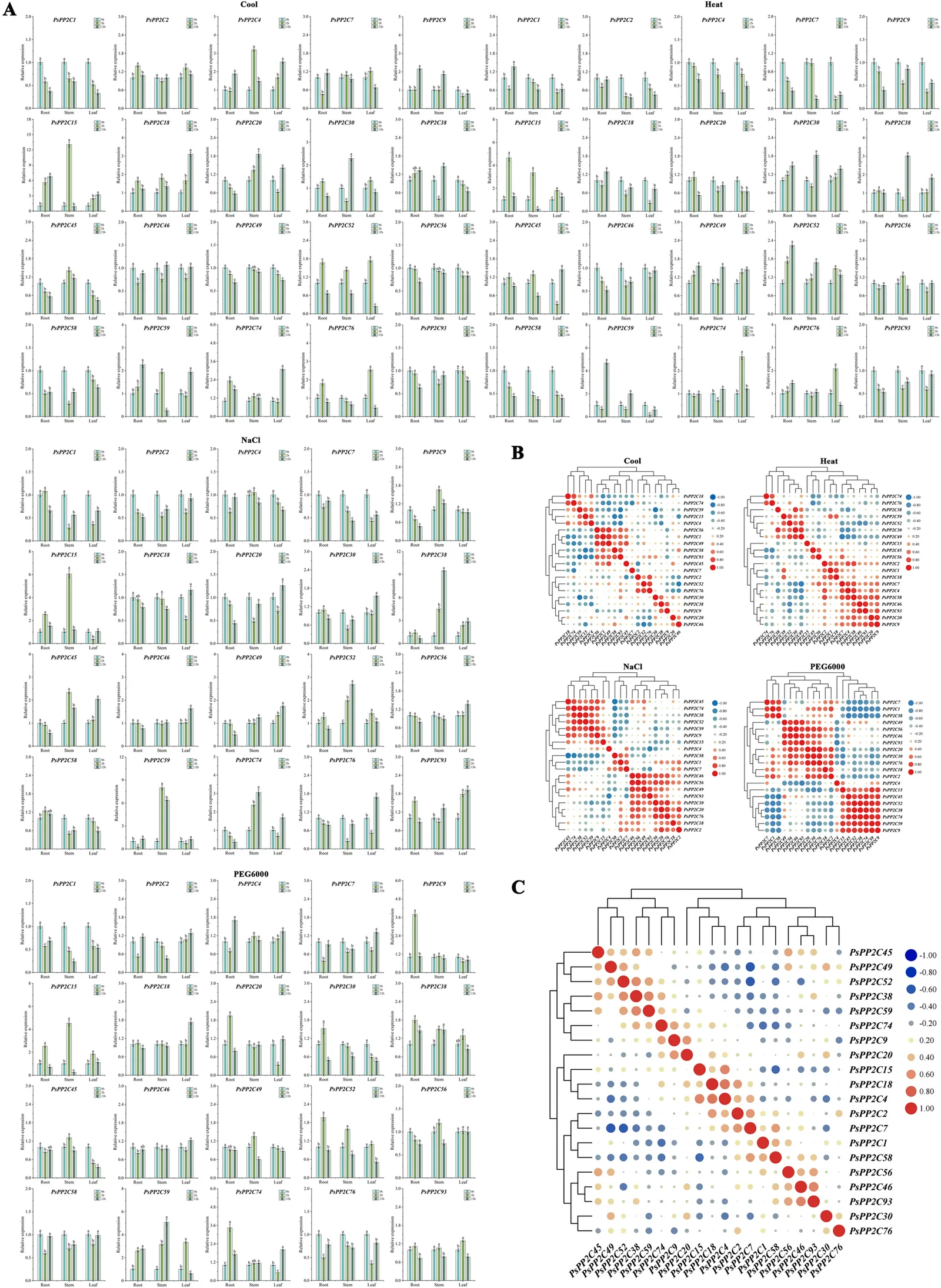
Tissue-specific expression patterns of *PsPP2C* genes under abiotic stress (**A**) Expression levels of 20 *PsPP2C* genes in the roots, stems, and leaves of pea seedlings under four abiotic stresses at 0 h, 2 h, and 24 h were detected using qRT-PCR; the lower-case letters above the columns indicate the differences between treatments (α = 0.05, LSD). **B** Gene expression correlation of 20 *PsPP2C* genes under one abiotic stress; the larger the labelled circle on the right side, the higher the correlation; red indicates a positive correlation and blue indicates a negative correlation. **C** Gene expression correlation of *PsPP2C* genes under four abiotic stresses; the larger the circle labelled on the right side, the higher the correlation; red indicates a positive correlation and blue, a negative correlation. qRT-PCR, Real-time quantitative reverse transcription polymerase chain reaction; LSD, Least significant difference

To investigate the role of the *PsPP2C* gene in peas under abiotic stress, quantitative real-time PCR (qRT-PCR) technology was used to analyze their expression patterns in different tissues of pea seedlings under four stress conditions: low temperature (4℃), high temperature (38℃), salt stress (NaCl), and drought stress (PEG6000) (Fig. 5A). Expression of *PsPP2C* genes was either induced or suppressed across all four stresses, with certain correlations observed among the expression profiles. For instance, *PsPP2C15* was significantly upregulated under all four abiotic stress conditions. Under cold stress (4 °C), its relative expression increased over time in roots, stems, and leaves, reaching a peak of 13-fold upregulation in stems. In contrast, under high-temperature stress (38 °C) and drought stress (PEG6000). Under salt stress (NaCl), its expression followed an increase-then-decrease pattern in roots and stems,, while in leaves it decreased initially and then gradually increased. By contrast, *PsPP2C1* expression was downregulated under all four abiotic stresses after 24 h.

To downregulated the expression correlations among *PsPP2C* genes under abiotic stress conditions, gene expression heatmaps were generated (Fig. 5B and C). Under the four stresses, strong positive or negative correlation clusters were evident among *PsPP2C* genes (Fig. 5B). Under drought stress, distinct positively correlated regions (including *PsPP2C9*, *PsPP2C15*, *PsPP2C38*, *PsPP2C45*, *PsPP2C52*, *PsPP2C59*, and *PsPP2C74*) and negatively correlated regions (including *PsPP2C1*, *PsPP2C7*, and *PsPP2C58*, along with some genes from the positive cluster) were identified (Fig. 5C). A limited number of gene pairs exhibited specific positive or negative correlations. For example, *PsPP2C7* showed a strong negative correlation with *PsPP2C49* and *PsPP2C52*, while *PsPP2C4* displayed a significant positive correlation with *PsPP2C15* and *PsPP2C18.*

### Expression pattern of the *PsPP2C* gene during pea grain development

**Fig. 6:**
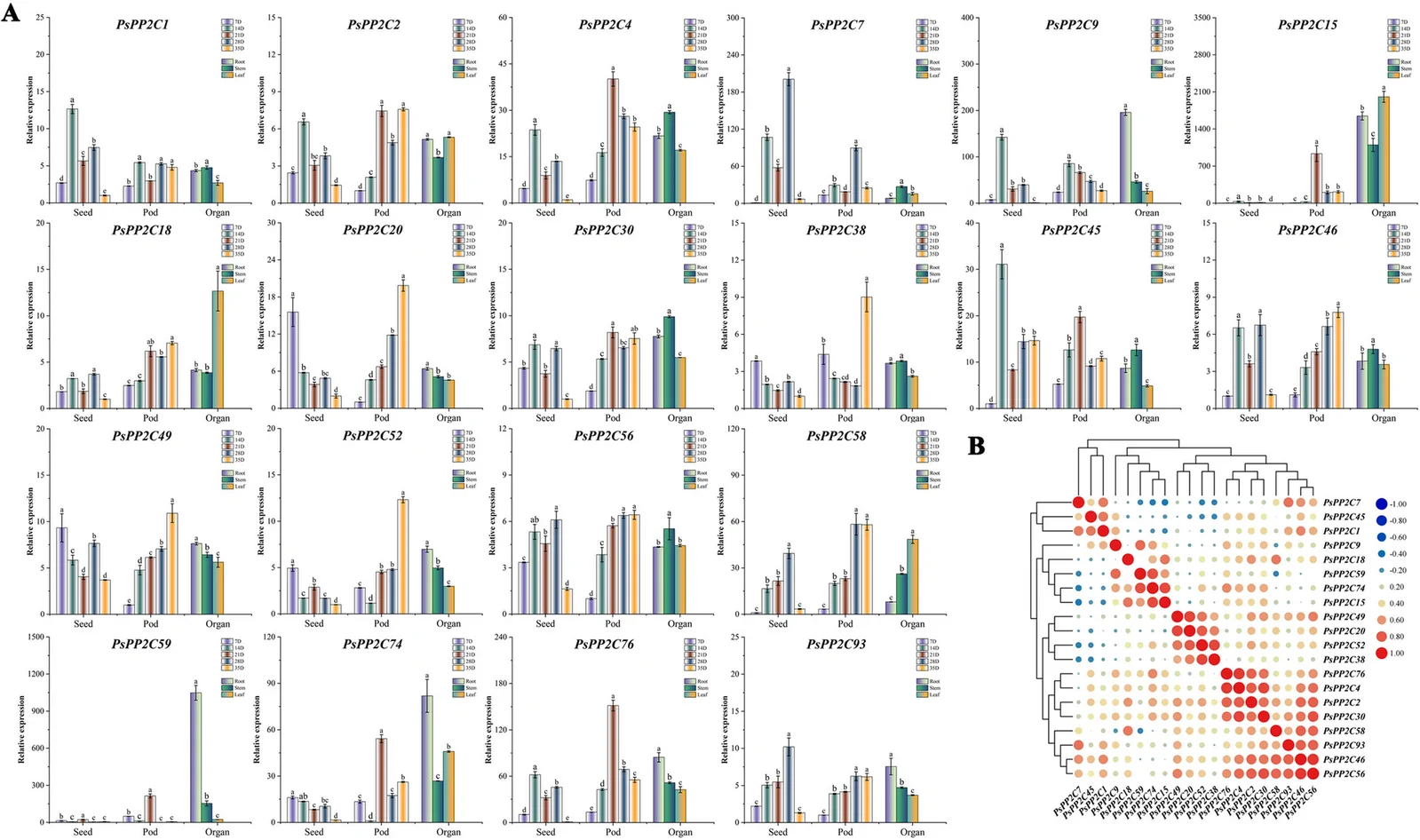
Expression pattern analysis of *PsPP2C* genes during pea grain development (**A**) Expression of 20 *PsPP2C* genes in seeds and pods (7, 14, 21, 28, and 35 d) during pea grain development, and in pea roots, stems, and leaves at 21 d was examined using qRT-PCR; lowercase letters above the columns indicate significant differences (α = 0.05, LSD). **B** Gene expression correlation of the 20 *PsPP2C* genes during grain development; the larger the labelled circle on the right side, the higher the correlation; orange is positive and green indicates a negative correlation

Pea seeds represent the primary economic product of the plant. To understand the potential role of *PsPP2C* genes in seed development, the gene expression patterns of these genes were analyzed in various plant tissues (seeds, pods, roots, stems, and leaves) across different stages of seed development (7, 14, 21, 28, and 35 d post-anthesis) (Fig. 6A). The results showed that relative expression of *PsPP2C* genes was generally upregulated during seed maturation. *PsPP2C15* was barely expressed during seed development but was highly expressed in pods at 21 d and in roots, stems, and leaves at 21 d. *PsPP2C59*, like *PsPP2C15* exhibited minimal expression in developing seeds but was highly expressed in pods at 21 d, as well as in roots, stems, and leaves at 21 d. Similarly, *PsPP2C59* showed low expression in seeds and was highly expressed in roots at 21 d. In contrast, *PsPP2C76* was mainly expressed during pod development. These findings suggest that *PsPP2C* genes play distinct role in the developmental processes of pea seeds and surrounding tissues.

Positive and negative correlations in expression were identified among *PsPP2C* genes during seed development (Fig. 6B). *PsPP2C7* showed negative correlations with *PsPP2C15*, *PsPP2C59*, and *PsPP2C74*. In contrast, *PsPP2C46* and *PsPP2C56* showed positive correlated with *PsPP2C4*, *PsPP2C2*, *PsPP2C30*, *PsPP2C58*, and *PsPP2C93*. Overall, the expression of most *PsPP2C* genes was positively correlated.

## Discussion

### Evolution of the pea *PP2C* gene family

The *PP2C* gene family plays an important role in plant hormone signaling, developmental processes, and abiotic stress responses [33, 34]. However, the *PP2C* gene family in pea has not been previously characterized. In this study, a systematic analysis of the pea *PP2C* gene family was performed, and the functions of 20 *PsPP2C* genes were investigated under four abiotic stresses conditions and during seed development. A total of 93 *PP2C* genes were identified in peas, and their physicochemical properties and subcellular localization were predicted (Table S1). All PsPP2C proteins are hydrophilic, with considerable variation in amino acid sequence, molecular weight, and isoelectric point (pI). Subcellular localization predictions indicated that most of *PsPP2C* proteins were located in the nucleus, cytoplasm, and chloroplasts, while a smaller number were predicted in the mitochondria, vesicles, and cytoskeleton. These findings suggest that diverse biological functions for PsPP2C proteins.

Gene amplification can lead to functional diversification and species differentiation, with DNA segment and tandem duplications serving as important mechanisms for gene family expansion and evolution [35]. In the pea *PP2C* family, four tandem duplication events and 23 DNA segment duplication events were identified (Table S4). Genes arising from duplication events exhibited structural similarity and shared conserved motifs. Among the 23 DNA segment duplications, one event (*PsPP2C52* and *PsPP2C68*) produced genes assigned to different subgroup, while all others remained within the same subgroup (F2 and I). Despite their different subgroup classifications, *PsPP2C52* and *PsPP2C68* displayed similar physicochemical properties, gene structures, and conserved motifs. All four tandem duplication events occurred on Chr3 and involved genes belonging to subgroup D. Subgroup D contained a total of four tandem and one DNA segment duplication event. Both duplication types contributed to the expansion of the pea *PP2C* gene family, with DNA segment duplications playing a greater overall role. However, within subgroup D specifically, tandem duplications contributed more to gene amplification than DNA segment duplications. Compared with tandem duplications, DNA segment duplications are more likely to preserve gene function [36]. Although *PsPP2C* gene derived from DNA segment duplication events showed high similarity in gene structure and conserved motifs, some divergence was observed in the retention of these gene copies. These genes may share similar biological functions, but whether they have diverged in function through evolution remains to be determined and warrants further investigation.

To examine phylogenetic relationship between the pea and other plant PP2C families, a phylogenetic tree was constructed using PP2C sequences from *A. thaliana* [31], *C. quinoa* [37], *C. sativus* [38], *O. sativa* [31], *T. aestivum* [29], and *Z. mays* [30]. Synteny analysis was also performed between pea and these species, as well as with three legume species: *G. max* [32], *C. arietinum*, and *A. hypogaea* [39] (Fig. 4; Table S5, S6). The phylogenetic tree revealed that most PsPP2C proteins clustered with PP2Cs from dicotyledonous plants and share highly similar conserved motifs. A subset of PsPP2C proteins formed separate branches yet retained high motif similarity with PP2Cs from other plants, suggesting these genes may have evolved distinct functions that contributed to pea growth and development.

Synteny analysis indicated that the pea PP2C family had the highest number of homologous gene pairs with other legume, followed by the monocotyledons and dicotyledons. Homologs of *PsPP2C* genes were identified in dicots, monocots and legumes; some were present only in dicots and legumes; and others were legumes -species. These results suggest that the ancestral *PsPP2C* gene likely prior to existed in the divergence of dicots and monocots lineages; Through environmental selection and species differentiation, some *PsPP2C* retained ancestral PP2C characteristics, while others evolved into legume-specific *PP2C* genes.

### Spatio-temporal expression of the *PsPP2C* gene family in response to four abiotic stressors during seed development

Numerous studies have demonstrated that members of the *PP2C* gene family are involved in various adaptive mechanisms regulating plant stress responses. For example, in rice, *OsPP2C09* was identified as a negative regulator of drought tolerance via ABA signaling [40]. Conversely, *OsPP2C27* regulates low-temperature tolerance in rice by modulating the OsMAPK3–OsHLH002–OsTPP1 pathway [41]. In *A. thaliana*, overexpression of *AtPP2CG1* enhanced salt tolerance, while its absence reduced tolerance to salt tolerance [42]. Additionally, overexpression of *AtPP2CF1* promoted inflorescence stem growth by activating cell proliferation, resulting in increased plant biomass [43].

The expression patterns of *PsPP2C* gene in different tissues of pea seedlings under abiotic stress conditions were analyzed, revealing both induction and suppression of expression under stress (Fig. 5). Under cold stress (4 °C), the relative expression of *PsPP2C15* in stems reached at 13 after 2 h and returned to baseline within 24 h. In contrast, under high-temperature stress (38 °C), expression of *PsPP2C59* in stems declined to 0.15 by 24 h. *PsPP2C15* was highly expressed under all four abiotic stresses, with peak expression occurring at 2 h before declining to or below baseline by 24 h. Notably, high expression of *PsPP2C15* was also observed in stems after 24 h. These findings suggest that *PsPP2C15* may function as a fast-response gene in peas, enabling quick accumulation under harsh environmental conditions to help mitigate stress-induced damage.

In contrast, the expression of *PsPP2C1* and *PsPP2C58* was suppressed under all four abiotic stressors, indicating they may act as negative regulators of pea stress resistance. The expression pattern of *PsPP2C4* exhibited opposite responses under high-temperature and cold stress: it was highly expressed in the roots, stems, and leaves under high temperature stress. This suggests that *PsPP2C4* may be an important regulatory gene in peas for responding to external temperature fluctuations.

Pea seeds are the primary edible and nutritional component of the plant [44]. During pea seed grain development, multiple *PsPP2C* genes showed extremely high expression, suggesting critical roles in this process (Fig. 6). *PsPP2C7* and *PsPP2C9* were highly expressed in seeds, while *PsPP2C15*, *PsPP2C59*, and *PsPP2C76* were highly expressed in pods. Notably, *PsPP2C9*, *PsPP2C15*, and *PsPP2C59* were highly also expressed in individual organs (roots, stems, and leaves). The expression of the *SlPP2C1* gene is increasing during the development of flowers and fruits in tomatoes, and when the expression of *SlPP2C1* is inhibited, fruit ripening is substantially accelerated [45]. *BjPP2C52* also acts as a regulatory factor in flower development in *Brassica juncea* [46]. *PsPP2C15* exhibited high expression under both abiotic stress and during seed development. Subcellular localization predictions revealed that PsPP2C15 was in chloroplasts. Additionally, multiple light-responsive cis-acting elements were identified in its promoter region, and subcellular localization predictions indicated chloroplast localization. These results imply that *PsPP2C15* may be an important regulatory gene involved in pea tissue growth and development, particularly in photosynthesis. Furthermore, cis-acting elements associated with ABA, methyl jasmonate, and salicylic acid responses were identified in the *PsPP2C15* promoter region. Given that the *PP2C* gene family is known to participate in ABA, MeJA, and SA signaling pathways [17, 18, 47], *PsPP2C15* may also function in these phytohormone-mediated processes. Therefore, *PsPP2C15* warrants further in-depth investigation.

## Conclusions

The pea *PP2C* gene family was analyzed on a genome-wide scale, leading to the identification of 93 *PsPP2C* genes, and designated *PsPP2C1*–*PsPP2C93* according to their chromosomal locations. The analysis included physicochemical properties, cis-acting elements, gene structure, gene duplication events, and evolutionary relationships of all 93 PsPP2C genes.

Gene amplification within the *PsPP2C* family predominantly through DNA segment duplication, leading to an expansion in member number. Expression patterns of *PsPP2C* were assessed via qRT-PCR in pea seedlings under four abiotic stress conditions, as well as in different seed tissues across developmental stages. The results indicate that *PsPP2C* genes are involved in responses to abiotic stress and seed development. Furthermore, numerous cis-acting elements associated with abiotic stresses and growth regulation were identified in *PsPP2C* promoter regions.

Based on these findings,, it is hypothesized that the pea PP2C family plays important roles in pea growth and development. This study highlights key candidate genes, such as *PsPP2C15*, which appear to regulate stress resistance and development. These genes provide precise targets for improving field pea varieties through gene editing or molecular marker-assisted breeding, with the aim of developing high-yielding, stress-resistant cultivars. The results thus offer valuable insight for future functional characterization of *PsPP2C* genes and for the genetic improvement of field pea.

## Materials and methods

### Pea material

The pea material used in this experiment was the early‑maturing pea variety “ZW6”, bred by the Institute of Animal Sciences, Chinese Academy of Agricultural Sciences. ZW6 has a growth period of approximately 66 days from emergence to maturity and is a widely cultivated variety in China, previously utilized for the construction of high-quality and high-resolution physical maps. Plants were grown in an artificial climate chamber at the College of Agriculture, Guizhou University. Pea seeds were sown in pots containing a 1:1 mixture of soil and vermiculite. The chamber was maintained at 75% relative humidity, 20 ± 1℃, with a 16 h light (25℃) 8 h dark photoperiod. After the 25 days, uniform, healthy seedlings free of pests and diseases were selected and exposed to four abiotic stresses: Salt (5% NaCl), Drought (30% PEG6000), Heat (38 °C), and Cold (4 °C). Root, stem, and leave samples (five biological replicates per time point) were collected at 0, 2, and 24 h after the treatment initiation. All samples were immediately frozen in liquid nitrogen and stored at -80 °C for subsequent use (five replicates per sample type).

### Identification and analysis of the PP2C family in pea

Pea gene and amino acid sequences were extracted based on genomic and genome annotation information [48]. Potential pea PP2C proteins were identified using BLASTp (score value ≥ 100, e value ≤ 1 × 10⁻¹⁰) by aligning sequences against known PP2C families from *Arabidopsis* and rice [49, 50], followed by removal of redundant sequences. A conserved Hidden Markov Model (HMM) for the PP2C domain (PF00481) was obtained from the Pfam Protein Family Database (http://pfam.sanger.ac.uk/), and the pea proteome was searched using HMMER 3.3.2 software with default parameters (http://hmmer.org/). All candidate PP2C proteins were subsequently examined using SMART (http://smart.emblheidelberg.de/) and CD-Search (https://www.ncbi.nlm.nih.gov/Structure/cdd/wrpsb.cgi) to confirm the presence of the PP2C domain. Sequences lacking the PP2C domains were removed, yielding the final set of pea PP2C genes. Subcellular localization was predicted using the WoLF PSORT (https://psort.hgc.jp/), Molecular weight (MW) and isoelectric point (pI) were obtained from the ExPasy web server (http://web.expasy.org/protparam/). Grand Average of Hydropathicity and Instability index (II) were calculated using TBtools software (https://github.com/CJ-Chen/TBtools).

### RNA extraction and qRT-PCR analysis of the pea

Pea tissue (0.1 g) was ground to fine powder in liquid nitrogen. Total RNA was extracted using the E. Z. N. A. Plant RNA Kit (Omega Bio-Tek, Inc., USA). RNA concentration and purity was assessed by spectrophotometry (OD_260/280_ ratio between 1.8 and 2.0, RIN value ≥ 7.0), and integrity was verified by 1% agarose gel electrophoresis. cDNA was synthesized from the RNA using the HiScript II Q RT Supermix for qPCR Kit. Twenty qRT-PCR primers pairs (Table S7) for the PsPP2C genes were designed with Premier 5.0 software; PsACT was used as the internal reference gene [51].

Quantitative real-time PCR (qRT-PCR) was performed using SYBR premix Ex Taq II (TaKaRa, Dalian, China) on a CFX96 real-time detection system (Bio-Rad, Hercules, California, USA). Each reaction included at least three technical replicates. Amplification was considered valid when the Ct value fell between 20 and 25, the amplification curve displayed normal kinetics, and the melting curve showed a single peak without nonspecific products. Gene expression levels were calculated using the 2^−(ΔΔCt)^ method [52]. Three biological replicates were analyzed for each sample.

### Classification and phylogeny of the *PsPP2C* family

The 93 *PsPP2C* genes were categorized into subgroups according to the established *Arabidopsis PP2C* family classification system. A phylogenetic tree was constructed using the neighbor-joining (NJ) method in Mega 11, with AtPP2C and PsPP2C proteins sequences, a bootstrap value of 1000, and default parameters for all other settings. Additionally, a separate phylogenetic tree was generated for PP2C proteins from six species: *A. thaliana*, *C. quinoa*, *C. sativus*, *O. sativa*, *T. aestivum*, and *Z. mays*.

### Gene structure and cis-acting elements of the *PsPP2C* gene family

Gene structure of the pea *PP2C* family was analyzed and visualized using the TBtools software (https://github.com/CJ-Chen/TBtools) by aligning PsPP2C genes against the pea genome [53]. Putative cis-acting elements located within the 2000 bp region upstream of each *PsPP2C* gene were predicted using the PlantCARE database (http://bioinformatics.psb.ugent.be/webtools/plantcare/html/) [54]. In addition, the ten most conserved motifs in PsPP2C proteins were identified using The MEME website (https://meme-suite.org/meme/tools/ MEME) [55].

### Distribution of *PsPP2C* genes on chromosomes and gene duplication

Based on the pea genome and gene annotation data, the physical positions of *PsPP2C* genes were extracted and mapped along chromosomes, with gene density visualized accordingly. Gene duplication events were identified using the Multiple Covariance Scanning Toolkit (MCScan X) with default parameters. Synteny analysis was performed and visualized with TBtools software (https://github.com/CJ-Chen/ TBtools), comparing *PsPP2C* genes against those from the following species: *A. thaliana*, *C. quinoa*, *C. sativus*, *O. sativa*, *T. aestivum*, *Z. mays*, *G. max*, *C. arietinum*, and *A. hypogaea*.

### Statistical analysis

Quantitative real-time PCR (qRT-PCR) data were analyzed by one-way analysis of variance (ANOVA) in JMP 6.0 software, followed by comparisons using the least significant difference method (LSD) test at a significance level of *p* < 0.05. Expression profiles of 20 *PsPP2C* genes under four abiotic stresses and during seed development were visualized as histograms using Origin software.

## Supplementary Information

Supplementary Material 1: Table S1: The 93 PsPP2C genes identified and protein sequences. Table S2: AtPP2C protein sequence for phylogeny. Table S3: The cis-acting element of the promoter sequence 2,000 bp upstream of 93 PsPP2C genes in Pisum sativum and conserved motifs of PsPP2C protein. Table S4: Tandem repeats and gene replication information of the PsPP2C gene. Table S5: PP2C protein sequences of six species and PP2C protein conserved motifs of seven species. Table S6: One-to-one orthologous relationships between Pisum sativum and nine different plants. Table S7: Primer sequences for real-time quantitative reverse transcription polymerase chain reaction.

## Data Availability

All data are included in the manuscript or its supplementary information files. The Complete pea (ZW6) genome sequence was obtained from the publicly available dataset published by Yang et al., [48]. Additional data requests may be directed to the corresponding author.
